# Long bone fractures in neonatal intensive care units of Afyonkarahisar: Five-year’s experience

**DOI:** 10.4274/tjod.galenos.2019.82346

**Published:** 2020-02-28

**Authors:** Mine Kanat Pektaş, Hilal Koyuncu, Afşin Ahmet Kundak

**Affiliations:** 1 Afyonkarahisar University of Health Sciences Faculty of Medicine, Department of Obstetrics and Gynecology, Afyonkarahisar, Turkey; 2 Afyonkarahisar University of Health Sciences Faculty of Medicine, Department of Child Health and Diseases, Afyonkarahisar, Turkey

**Keywords:** Femoral fracture, intensive care units, metabolic bone diseases, newborn

## Abstract

**Objective:**

To determine the incidence of long bone fractures and the clinical features related with these fractures diagnosed in neonatal intensive care units (ICUs) within the province of Afyonkarahisar in Turkey.

**Materials and Methods:**

The incidence of clavicular fractures was 2.4 in 1000 live births, and the incidence of femoral fractures was 0.32 in 1000 live births at the neonatal ICUs of Afyonkarahisar.

**Results:**

The incidence of birth trauma-related femoral fracture was 0.16 in 1000 live births, and the incidence of femoral fractures related with osteopenia of prematurity was 1.08 in 1000 live births. The mean gestational age at delivery was 39 weeks, the mean birth weight was 3.308 grams, and the male/female ratio was 3:2 for newborns with birth trauma-related femoral fractures. The mean gestational age at delivery was 30.4 weeks, the mean birth weight was 1256 grams, and the male/female ratio was 2:3 for newborns who had femoral fractures related with osteopenia of prematurity. Breech presentation was present in three newborns (60%), and cesarean section was the type of delivery in all newborns with birth trauma-related femoral fractures.

**Conclusion:**

Cesarean delivery does not reduce the risk for birth trauma-associated femoral fractures, and there is a risk for femoral fracture in cases of emergency cesarean performed for malpresentation. In order to overcome osteopenia of prematurity, calcium, phosphorus, and vitamin D should be supplemented in premature newborns with intrauterine growth retardation and receive long-term total parenteral nutrition.

**PRECIS:** Neonatal fractures at intensive care.

## Introduction

Bone fractures are rarely encountered in newborns^(1)^. The incidence of clavicular fractures is about 2 to 3.5 in 1000 live births, whereas the incidence of femoral fracture is 0.13 in 1000 live births^(1,2)^. Long bone fractures of newborns might occur as a consequence of vaginal delivery-related trauma^(2)^. Moreover, newborns treated in intensive care units (ICUs) have an increased risk for long bone fractures due to prematurity, low birth weight, and the administration of pharmacologic agents^(3)^. The incidence of long bone fractures ranges between 1.2% and 10.5% in neonatal ICUs^(2,4)^. This study aims to specify the incidence of long bone fractures and the clinical characteristics related with these fractures in newborns treated in three ICUs within the province of Afyonkarahisar in Turkey.

## Materials and Methods

A total of 54 clavicular fractures and 10 femoral fractures were diagnosed in 31,058 live births at Afyonkarahisar University of Health Sciences Hospital, Afyonkarahisar State Hospital, and Afyonkarahisar Private Park Life Hospital between 2014 and 2019. The incidence of clavicular fracture was 2.4 in 1000 live births, and the incidence of femoral fractures was 0.32 in 1000 live births. Five cases of femoral fractures were related with birth trauma, whereas the remaining five cases were associated with osteopenia of prematurity. Thus, the incidence of birth trauma-related femoral fracture was 0.16 in 1000 live births, and the incidence of femoral fractures related with osteopenia of prematurity was 1.08 in 1000 live births. This is a retrospective review of ten newborns who were diagnosed as having femoral fractures. Data related with perinatal characteristics, treatment procedures, and administered drugs in the ICUs were recorded. Femoral fractures related with birth trauma were defined as fractures that occurred during delivery and were found to be unrelated with postnatal trauma. The remaining femoral fractures were identified as fractures related with osteopenia of prematurity.

## Results

Table 1 shows the clinical characteristics of five newborns who were diagnosed as having birth trauma-related femoral fractures and five newborns who had femoral fractures related with osteopenia of prematurity. For the newborns with birth trauma- related femoral fractures, the mean gestational age at delivery was 39 weeks, the mean birth weight was 3308 grams, and the male/female ratio was 3 (60.0%): 2 (40.0%). Breech presentation was present in three newborns (60%) and cesarean section was the type of delivery in all newborns with birth trauma-related femoral fractures. All of these fractures were diagnosed within the first day of life due to the irritability of the newborn and immobility of the involved extremity. For newborns that had femoral fractures related with osteopenia of prematurity, the mean gestational age at delivery was 30.4 weeks, the mean birth weight was 1256 grams, and the male/female ratio was 2 (40.0%): 3 (60.0%). Intrauterine growth retardation (IUGR) was specified in three newborns (60%), bronchopulmonary dysplasia (BPD) was detected in three newborns (60%), and nosocomial sepsis was diagnosed in two newborns (40%) who were diagnosed as having osteopenia of prematurity.

Case #9 was one of the dichorionic twins who had Arnold Chiari type 2 malformation and myelomeningocele. He was diagnosed as having bilateral femoral fractures related with osteopenia of prematurity. The mean duration of hospitalization was 80 days, the mean duration of mechanical ventilation was 38.4 days, and the mean time of diagnosis was 46.4 days for all newborns with femoral fractures related with osteopenia of prematurity. Five newborns with femoral fractures were treated with traction, and three were treated with splinting; the remaining two newborns were treated with both traction and splinting. Complete improvement was noted in 4 weeks for all newborns except one who was lost to follow-up.

## Discussion

The incidence of clavicular fractures has been reported as about 2 to 3.5 in 1000 live births, whereas the incidence of femoral fracture has been reported as 0.13 in 1000 live births^(1,2)^. A Portuguese study detected one or more fractures in 1.1% of neonates who were admitted to the ICU. The most common fracture was clavicle fracture in 60 newborns (79%), followed by skull fracture in 6 newborns (8%) in that study^(5)^. Similarly, a Welsh study estimated the incidence of fractures as 1.6% for neonatal ICUs. The fracture sites included ribs (n=45), humerus (n=5), ulna (n=3), radius (n=4), femur (n=8), tibia (n=1), clavicle (n=4), and skull (n=1)^(1)^. As for the present study, the incidence of clavicular fracture was 2.4 in 1000 live births, and the incidence of femoral fractures was 0.32 in 1000 live births. Birth trauma-related bone fractures have been defined as fractures that occur during the first week of life and which are found to be unrelated with postnatal trauma^(6)^. Malpresentation, preterm delivery, fetal macrosomia, multiple pregnancy, metabolic bone diseases, and emergency cesarean delivery have been identified as the risk factors for birth trauma^(7)^. Birth trauma-related bone fractures usually appear as a result of the maneuvers performed for breech presentation in vaginal deliveries^(6,7)^. In the event of malpresentation, cesarean delivery is performed, which has significantly decreased perinatal morbidity and mortality^(8)^. However, birth trauma occurs in both vaginal and cesarean deliveries, and preferring cesarean section over vaginal delivery does not eliminate the risk of birth trauma^(9)^. In fact, sudden and careless traction of the newborn’s extremities and insufficient myometrial relaxation might cause birth trauma-related bone fractures^(8,9)^. Hannah et al.^(10)^ reported the incidence of long bone fractures as 0.5% for vaginal deliveries and 0.1% for cesarean deliveries in cases of breech presentation. On the contrary, cesarean deliveries were associated with a significantly higher incidence of long bone fractures than vaginal deliveries in pregnancies with breech presentation^(11,12,13,14,15)^. In this study, the incidence of birth trauma-related femoral fracture was 0.16 in 1000 live births. In this study, all newborns with birth trauma-related femur fractures were delivered by cesarean section. The indications for cesarean delivery were malpresentation (breech presentation) in three cases (60%), dystocia in one case (20%), and fetal distress in one case (20%). Two cesarean deliveries performed for dystocia and fetal distress were emergency deliveries. Basha et al.^(11)^ claimed that birth trauma-related bone fractures were diagnosed within a mean time period of 1.5 days. Morris et al.^(16)^ stated that birth trauma-related femoral fractures were diagnosed within a mean time period of 6.3 days. A high index of suspicion may help to make an early diagnosis in newborns that have risk factors for bone fractures because bone fracture-related symptoms may be noticed relatively late^(11,16)^. In this study, all newborns with birth trauma-related femur fractures were diagnosed within the first day of life. Osteopenia of prematurity is also known as a metabolic bone disease of prematurity. This clinical entity is specified when postnatal bone mineralization is significantly lower than intrauterine bone mineralization adjusted for gestational age. The incidence for osteopenia of prematurity increases as gestational age and birth weight decrease^(17)^. Wei et al.^(1)^reported that the neonates who had non-traumatic fractures had significantly lower gestational age and birth weight. Osteopenia, need for multiple medical interventions, and late diagnosis of fractures were significantly more frequent in neonates who had non-traumatic fractures. Osteopenia of prematurity usually appears between the 6^th^ to 12^th^ weeks of corrected gestational age. This clinical entity affects 20% to 30% of newborns weighing less than 1500 grams and up to 50% to 60% of newborns weighing less than 1000 grams^(5,17)^. Although its exact incidence is unknown, bone fractures related with osteopenia of prematurity appear in 1.2% to 10% of very-low-birthweight newborns^(18,19)^. In this study, the incidence of osteopenic femoral fractures was 1.08 in 1000 live births.

Preterm delivery; IUGR; necrotizing enterocolitis; insufficient intake of calcium, phosphorus and vitamin D; and long-term administration of total parenteral nutrition (TPN) and calcium and phosphorus losing drugs (diuretics, steroids and caffeine) have all been addressed as the risk factors for osteopenia of prematurity^(20)^. Preterm delivery interrupts this accumulation and induces osteopenia of prematurity because the majority of fetal calcium and phosphorus accumulation takes place in the last trimester^(20)^. All of the newborns with femur fractures related with osteopenia of prematurity were born before the 37^th^ gestational week. The administration of TPN over two weeks has been identified as a risk factor for osteopenia in premature newborns with very low birth weight^(21)^. Three out of five newborns who had femur fractures related with osteopenia of prematurity had received TPN treatment (60%). Caffeine use triggers osteopenia of prematurity by inducing demineralization and calciuria^(18,20)^. Four out of five newborns who had femur fractures related with osteopenia of prematurity had a history of caffeine intake (80%). BPD also leads to osteopenia of prematurity by inducing demineralization^(22)^. BPD was detected in three out of five newborns with femur fractures related with osteopenia of prematurity (60%). The existence of IUGR has been denoted as a factor that independently increases the risk for osteopenia of prematurity by ten times^(23)^. This finding can be attributed to the significantly lower maternal and fetal calcium intake, significantly higher serum parathormone levels, and significantly lower vitamin D levels in umbilical cord blood^(24,25,26,27)^. Accordingly, IUGR was determined in three out of five newborns who had femur fractures related with osteopenia of prematurity (60%).

Osteopenia of prematurity usually presents with non-specific clinical symptoms. There are no specific criteria for diagnosing osteopenia of prematurity. Several biochemical markers have frequently been used as screening tools and diagnostic markers, but threshold values vary widely. Standard X-ray is generally used to diagnose osteopenia of prematurity, but this method cannot detect osteopenia unless bone mineralization is reduced by 20%^(28)^. Femoral fractures of newborns can be treated with either traction or splinting^(15)^. In this study, five newborns with femoral fractures were treated with traction, three newborns were treated with splinting, and two newborns had a combination of splinting and traction. When treated, neonatal femur fractures have an excellent prognosis^(15)^. Thus, all newborns in this study improved without any sequelae.

### Study Limitation

The power of the present study is limited by its retrospective design, relatively small cohort size, and the lack of data related to the long bone fractures diagnosed in newborns who were admitted to outpatient clinics.

## Conclusion

Malpresentation, preterm delivery, fetal macrosomia, multiple pregnancy, metabolic bone diseases, and emergency cesarean delivery have been defined as the risk factors for birth trauma-related bone fractures. Birth trauma occurs in both vaginal and cesarean deliveries and preferring cesarean section over vaginal delivery does not eliminate the risk of birth trauma. Preterm delivery, IUGR, necrotizing enterocolitis, insufficient intake of calcium, phosphorus and vitamin D, long-term administration of TPN and calcium and phosphorus losing drugs (diuretics, steroids and caffeine) have all been addressed as the risk factors for osteopenia of prematurity. In order to overcome osteopenia of prematurity, calcium, phosphorus, and vitamin D should be supplemented in premature newborns that have IUGR and receive long-term TPN.

## Figures and Tables

**Table 1:**
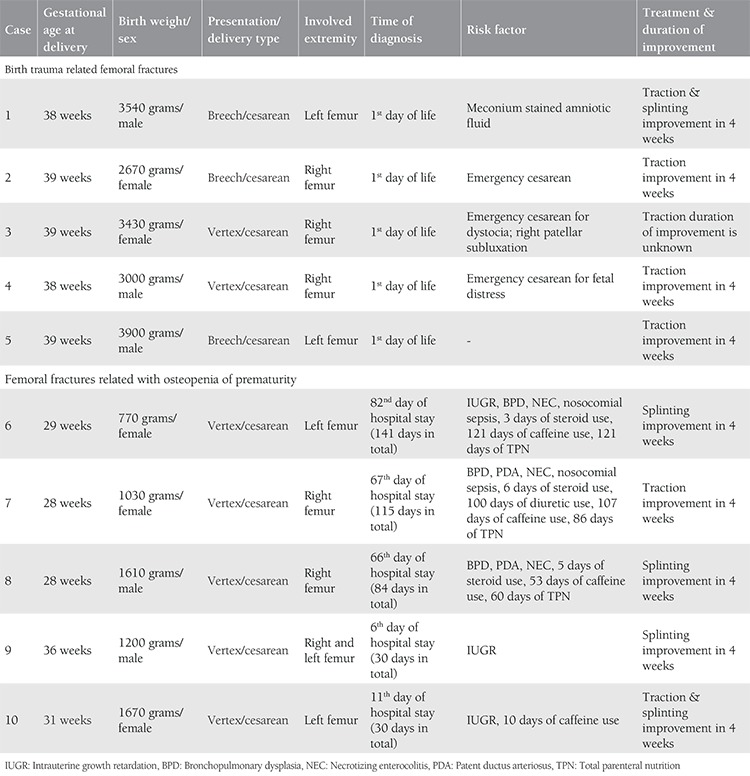
Birth trauma and prematurity related femoral fractures

## References

[1] Wei C, Stevens J, Harrison S, Mott A, Warner J (2012). Fractures in a tertiary neonatal intensive care unit in Wales. Acta Paediatr.

[2] Bülbül A, Okan F, Nuhoğlu A (2006). Yenidoğanın fiziksel doğum travmaları. Haseki Tıp Bülteni.

[3] Faienza MF, D’Amato E, Natale MP, Grano M, Chiarito M, Brunetti G, et al (2019). Metabolic Bone Disease of Prematurity: Diagnosis and Management. Front Pediatr.

[4] Caviglia H, Garodo CP, Palazzi FF, Meana NV (2005). Pediatric fractures of the humerus. Clin Orthop Relat Res.

[5] Machado A, Rocha G, Silva AI, Alegrete N, Guimaraes H (2015). Bone fractures in a neonatal intensive care unit. Acta Med Port.

[6] Dias E (2012). Bilateral fracture following birth trauma. J Clin Neonatol.

[7] Swedish Collaborative Breech Study Group (2005). Term breech delivery in Sweden: mortality relative to fetal presentation and planned mode of delivery. Acta Obstet Gynecol Scand.

[8] Li T, Rhoads GG, Smulan J, Demisic K, Wartenberg D, Kruse L (2003). Physician Cesarean Delivery Rates and Risk Adjusted Perinatal Outcomes. Obstet Gynecol.

[9] Matsubara S, Izumi A, Nagai T, Kikkawa I, Suzuki F (2008). Femur fracture during abdominal breech delivery. Arch Gynecol Obstet.

[10] Hannah ME, Hannah WJ, Hewson SA, Hodnett ED, Saigal S, Willian AR (2000). Planned caesarean section versus planned vaginal birth for breech presentation at term: a randomised multicentre trial. Lancet.

[11] Basha A, Amarin Z, Abu-Hassan F (2013). Birth-associated long-bone fractures. Int J Gynaecol Obstet.

[12] Canpolat FE, Köse A, Yurdakök M (2010). Bilateral humerus fracture in a neonate after cesarean delivery. Arch Gynecol Obstet.

[13] Cebesoy FB, Cebesoy O, Incebıyık A (2009). Bilateral femur fracture in a newborn: An extreme complication of cesarean delivery. Arch Gynecol Obstet.

[14] Garcia Garcia IE, de la Vega A, Garcia Pragosol I (2002). Long bone fractures in extreme low birth weight infants at birth: obstetrical considerations. P R Health Sci J.

[15] Toker A, Perry ZH, Cohen E, Krymko H (2009). Cesarean section and the risk of fractured femur. Isr Med Assoc J.

[16] Morris S, Cassidy N, Stephens M, McCormack D, McManus F (2002). Birth associated femoral fractures: incidence and outcome. J Pediatr Orthop.

[17] Ukarapong S, Venkatarayappa SKB, Navarrete C, Berkovitz G (2017). Risk factors of metabolic bone disease of prematurity. Early Hum Dev.

[18] Ali E, Rockman-Greenberg C, Moffatt M, Narvey M, Reed M, Jiang D (2018). Caffeine is a risk factor for osteopenia of prematurity in preterm infants: a cohort study. BMC Pediatr.

[19] Chen W, Yang C, Chen H, Zhang B (2018). Risk factors analysis and prevention of metabolic bone disease of prematurity. Medicine (Baltimore).

[20] Abrams SA;, Comittee on Nutrition (2013). Calcium and vitamin D requirements of enterally fed preterm infants. Pediatrics.

[21] Rustico SE, Calabria AC, Garber SJ (2014). Metabolic bone disease of prematurity. J Clin Transl Endocrinol.

[22] Harrison CM, Johnson K, McKechnie E (2008). Osteopenia of prematurity: a national survey and review of practice. Acta Paediatr.

[23] Montaner Ramón A, Fernández Espuelas C, Calmarza Calmarza P, Rite Gracia S, Oliván Del Cacho MJ (2017). Risk factors and biochemical markers in metabolic bone disease of premature newborns. Rev Chil Pediatr.

[24] Namgung R, Tsang RC (2000). Factors affecting newborn bone mineral content: in utero effects on newborn bone mineralization. Proc Nutr Soc.

[25] Robinson CJ, Wagner CL, Hollis BW, Baatz JE, Johnson DD (2011). Maternal vitamin D and fetal growth in early-onset severe preeclampsia. Am J Obstet Gynecol.

[26] Barrera D, Diaz L, Noyola-Martinez N, Halhali A (2015). Vitamin D and Inflammatory Cytokines in Healty and Preeclamptic Pregnancies. Nutrients.

[27] Olmos-Ortiz A, Avila E, Durand-Carbajal M, Diaz L (2015). Regulation of calcitriol biosyntesis and activity: Focus on gestastional vitamin D deficiency and adverse pregnancy outcomes. Nutrients.

[28] Mannan MA, Jahan I, Rahman MZ, Hasan Z, Dey AC, Shahidullah M (2015). Osteopenia of Prematurity: Are We at Risk?. Mymensingh Med J.

